# Has the 8th American joint committee on cancer TNM staging improved prognostic performance in oral cancer? A systematic review

**DOI:** 10.4317/medoral.25983

**Published:** 2024-02-18

**Authors:** María C Erazo-Puentes, Alba Sánchez-Torres, José M Aguirre-Urizar, Javier Bara-Casaus, Cosme Gay-Escoda

**Affiliations:** 1 DDS. Fellow of Master of Oral Surgery and Orofacial Implantology. School of Medicine and Health Sciences, University of Barcelona, Spain; 2 DDS, MS, PhD. Master of Oral Surgery and Orofacial Implantology. Associate Professor of the Oral Surgery Department, School of Medicine and Health Sciences, University of Barcelona, Spain; 3 MD, DDS, MS, PhD. Department of Stomatology. University of the Basque Country / EHU, Leioa, Spain; 4 MD, PhD, OMFS. Director of the Maxillofacial Institute of Sagrat Cor University Hospital. Co-Director of the Specialist Course in TMJ and Orofacial Pain, University of Barcelona. Director of the Postgraduate Course in Oral Medicine and Surgery of the Catalan Society of Odontology and Stomatology, Barcelona, Spain; 5 MD, DDS, MS, PhD, EBOS, OMFS. Former Chairman and Professor of the Oral and Maxillofacial Surgery Department, School of Dentistry, University of Barcelona. Director of the Master Degree Program in Oral Surgery and Implantology (EFHRE International University / FUCSO). Coordinator/Researcher of the IDIBELL Institute. Head of the Oral and Maxillofacial Surgery and Implantology Department, Teknon Medical Center, Barcelona, Spain

## Abstract

**Background:**

The 8th edition of the American Joint Committee on Cancer (AJCC) classification has introduced two new parameters: depth of invasion (DOI) and extranodal extension (ENE). The aim of this systematic review was to determine whether this 8th edition referred to oral squamous cell carcinoma (OSCC) offers performance superior to that of the 7th edition in relation to overall survival (OS) and disease-specific survival (DSS).

**Material and Methods:**

The review was carried out following the PRISMA (Preferred Reporting Items for Systematic Reviews and Meta-analyses) guidelines. The PubMed (MEDLINE), Scopus and Cochrane Library databases were searched covering the period up until April 7th, 2022.

**Results:**

Thirteen retrospective cohort studies were finally included. The introduction of DOI and ENE in the 8th edition of the AJCC classification resulted in improved prognostic performance of the classification.

**Conclusions:**

Patients with OSCC can be better classified in relation to OS and DSS, while maintaining the simplicity and ease of use of the classification. This allows more appropriate treatment protocols to be applied and affords a better estimation of the prognosis of each patient.

** Key words:**TMN Staging, AJCC8, squamous cell carcinoma, oral cancer, prognosis.

## Introduction

Lip, oral cavity and pharyngeal cancers were responsible for 292,300 deaths worldwide in 2012, accounting for 3.8% of all cancers and 3.6% of all cancer deaths (1). Similar Figures were subsequently published in 2018 (3.1% and a 2.8 %, respectively) (2). The incidence of these tumors may vary depending on variables such as geographical location, sex and age of the patients, and the presence of specific toxic habits such as betel consumption (1).

More than 90% of all oral cavity cancers are squamous cell carcinomas (OSCCs) and present a mortality rate of close to 50%. The most frequent location is the tongue (3). Other less frequent malignancies of the oral cavity are lymphomas, malignant salivary neoplasms, sarcomas, malignant odontogenic neoplasms and melanomas (4).

The 8th edition of the American Joint Committee on Cancer (AJCC) tumor staging manual, published in 2017 (5), is an abridgement of all available information on adult cancer staging for all relevant anatomical locations. The TNM classification plays an important role in the clinical study, research, registration and control of cancer. It has been widely used, because it helps to determine the prognosis, plan treatment better, and evaluate the results after treatment (6).

The most important changes in the 8th edition of the AJCC classification referred to oral cancer are the addition of depth of invasion (DOI) and extranodal extension (ENE) as modifying factors for categories T and N, respectively, and the elimination of classification T0 (7). This new manual emphasizes the importance of distinguishing between tumor thickness and DOI, and stipulates the use of this parameter to improve the prognostic accuracy of T staging in OSCC. Specifically, DOI assesses the invasiveness of OSCC independently of any exophytic or ulcerative components. This factor was not contemplated in the 7th edition of the AJCC classification, and was finally included in the current edition due to consensus-based agreement that tumors of greater thickness or DOI are associated to a higher risk of nodal metastasis and to poorer survival outcomes (8). On the other hand, ENE is defined as the extension of metastatic carcinoma in the lymph node through its capsule and into the surrounding connective tissue, regardless of the existence of an associated stromal reaction (5). This factor has also been added in the new edition, since it has been related to a poorer prognosis in patients with OSCC (9).

The purpose of this systematic review was to assess the prognostic performance of the 8th edition of the AJCC classification compared to the 7th edition, in relation to overall survival (OS) and disease-specific survival (DSS).

## Material and Methods

This systematic review adheres to the Preferred Reporting Items for Systematic Reviews and Meta-Analyses (PRISMA) guidelines (10).

The aim of the study was defined through the following PICO (patient intervention comparison outcome) question: “In patients diagnosed with OSCC, does the 8th edition of the AJCC classification offer better prognostic performance in relation to 5-year overall survival (OS) and disease-specific survival (DSS) than the 7th edition?”

The inclusion criteria were: randomized clinical trials and prospective or retrospective cohort studies comparing both editions in classifying patients diagnosed with OSCC. Studies in which the data extraction was not possible, studies including less than 10 patients, case series and case reports were excluded. No language or publication date restrictions were applied.

Two independent reviewers (M.C.E.P., A.S.T.) carried out an electronic search of the PubMed (MEDLINE), Scopus and Cochrane Library databases covering the period up until April 7th, 2022. The search strategy used was: (("OSCC"[All Fields] OR ((("mouth"[MeSH Terms] OR "mouth"[All Fields]) OR "oral"[All Fields]) AND ((("carcinoma, squamous cell"[MeSH Terms] OR (("carcinoma"[All Fields] AND "squamous"[All Fields]) AND "cell"[All Fields])) OR "squamous cell carcinoma"[All Fields]) OR (("squamous"[All Fields] AND "cell"[All Fields]) AND "carcinoma"[All Fields])))) AND (("7th"[All Fields] OR ("8th"[All Fields] AND (("edition"[All Fields] OR "edition s"[All Fields]) OR "editions"[All Fields]))) OR ("am j clim change"[Journal] OR "ajcc"[All Fields]))) AND ((((((((((("mortality"[MeSH Subheading] OR "mortality"[All Fields]) OR "survival"[All Fields]) OR "survival"[MeSH Terms]) OR "survivability"[All Fields]) OR "survivable"[All Fields]) OR "survivals"[All Fields]) OR "survive"[All Fields]) OR "survived"[All Fields]) OR "survives"[All Fields]) OR "surviving"[All Fields]) OR (("prognosis"[MeSH Terms] OR "prognosis"[All Fields]) OR "prognoses"[All Fields])).

A manual search was made of articles published during the last 5 years in the following journals: Head and Neck, Oral Oncology, International Journal of Clinical Oncology, Journal of Clinical Oncology, International Journal of Cancer and Annals of Surgical Oncology. In addition, the references of the included studies were checked in order to detect all relevant articles.

The selection of the studies was initially made by reading the titles and abstracts, followed by full-text assessment to decide final eligibility. A third author (C.G.E.) resolved any disagreement. The inter-examiner agreement was calculated by means of Cohen's kappa coefficient (κ). The Newcastle-Ottawa Scale (NOS) (11) was used to assess the quality of the included studies. Finally, data extracted from the included studies were synthesized in Table format.

## Results

**Figure 1:**
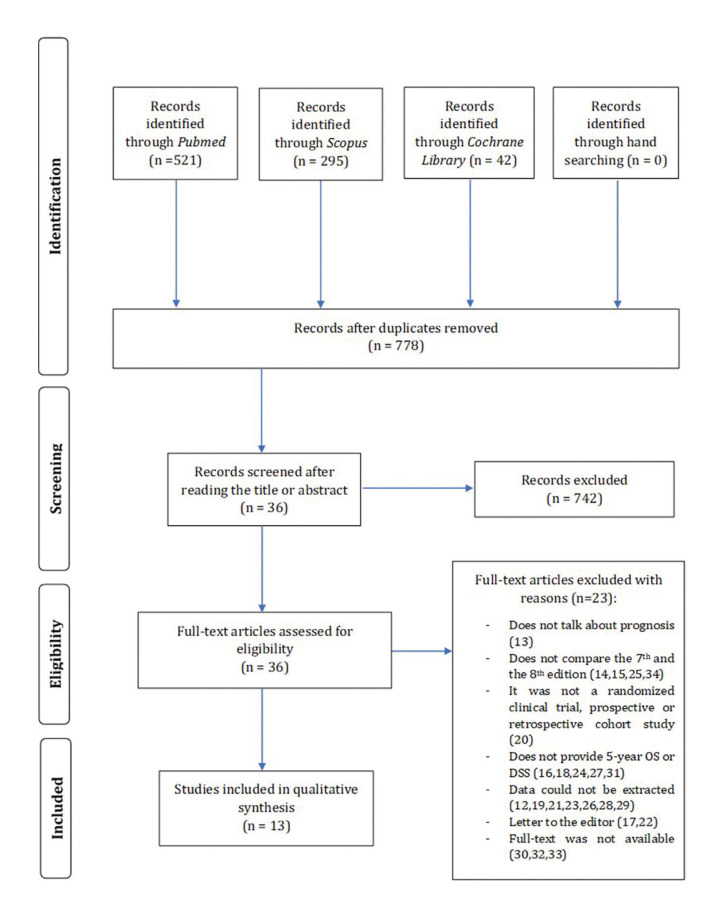
Flow diagram of the screening process of articles for the review.

From the initial 778 articles identified by the electronic search, 36 studies were selected for full-text assessment. Of these, 23 studies (12-34) were excluded due to the reasons shown in the flowchart (Fig. 1), which reflects the selection process of the studies during the systematic review. Thirteen articles (35-47) were finally included in the qualitative analysis, all of them being retrospective cohort studies. No additional articles were included from the manual search. Cohen’s kappa coefficient was 0.8045. According to the NOS scale (11), all the included articles were rated as being of high quality (35-47).

Table 1 shows the analyzed studies and the characteristics of the participants. Six studies were conducted at university hospitals (35,38,43,46,47), one in a private hospital (37), two in public or non-profit hospitals (44,45), one in both public and private hospitals (48), and three of them did not specify where the study was carried out (41,42,46). Of the total 85,434 patients, 52,082 (61%) were male and 33,352 (39%) females. In terms of location, 26,548 (31.1%) cases were located on the tongue; 9,277 (10.9%) on the gingiva, alveolar ridge or retromolar trigone; 9,638 (11.3%) on the floor of the mouth; 4,010 (4.7%) on the buccal mucosa; 1,707 (4.7%) on the hard palate; 4,821 (5.6%) on the lips, and 1,712 (2%) in other locations - though not all studies cited the affected location. The treatment of patients was likewise not cited by all authors. A total of 59,743 (69.9%) patients were treated by surgery alone; 16,945 (19.8%) received surgical treatment and adjuvant treatment in the form of radiotherapy, chemotherapy or chemoradiotherapy; and 4,981 (5.8%) patients received chemotherapy, radiotherapy, chemoradiotherapy or brachytherapy alone. The follow-up time was highly variable and difficult to compare between studies, as shown in Table 1.

**Table 1:**
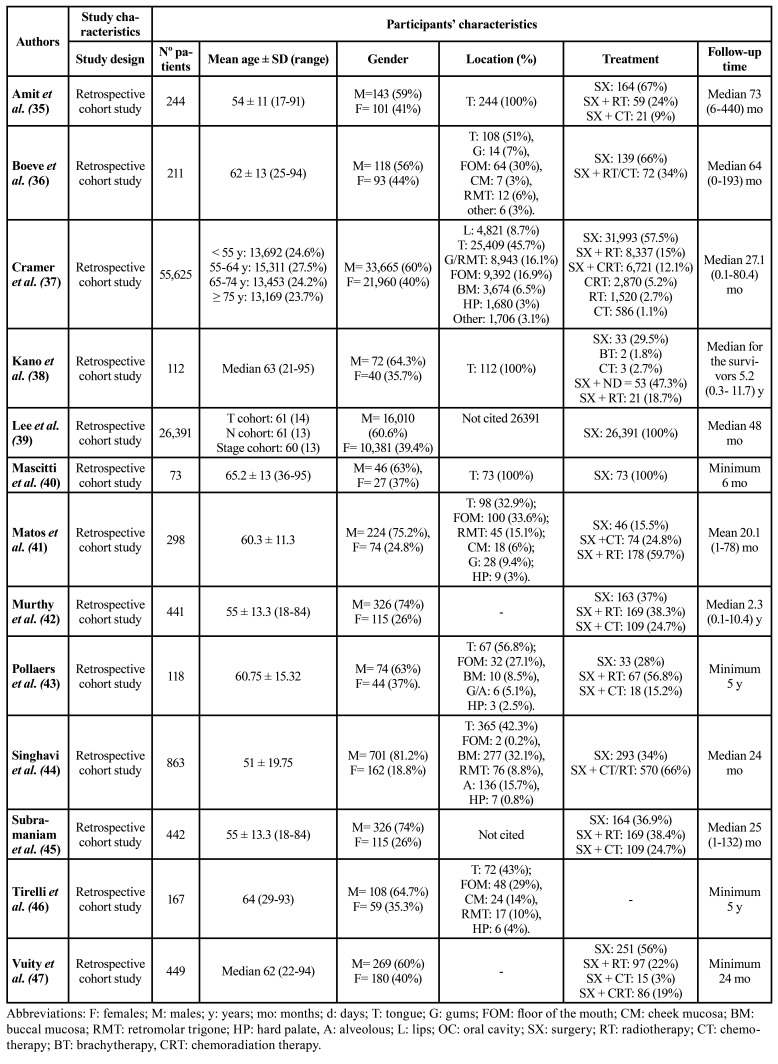
Study and participants’ characteristics. All are retrospective cohort studies.

Table 2 and Table 3 show the OS and DSS of patients included by T and N classification according to the 7th and 8th editions of the AJCC classification. On comparing OS in patients who remained in the same T or N classification versus those who were upstaged to another classification, several studies (35,40,41) reported poorer OS and DSS (35,41) among patients classified at a higher classification. In contrast, Lee *et al*. (39) recorded an increase in OS in most groups. On the other hand, studies comparing classifications separately found similar or better data referred to OS and DSS in T and N classifications in the 8th edition compared to the 7th edition (36,38,42,45-47).

**Table 2:**
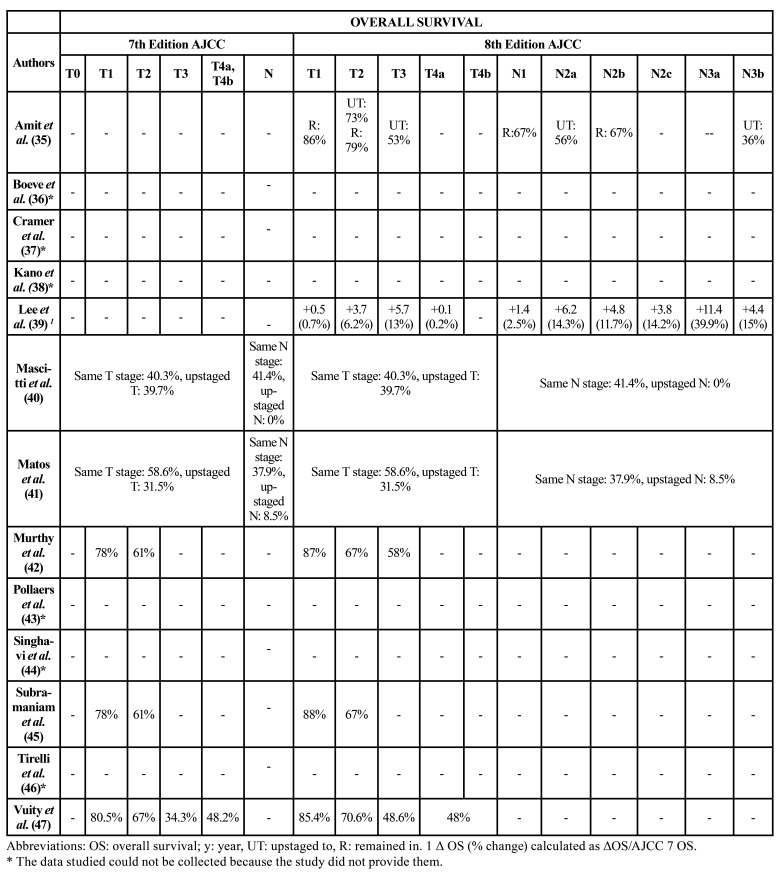
5-year overall survival by T and N 7th and 8th edition AJCC groups.

**Table 3:**
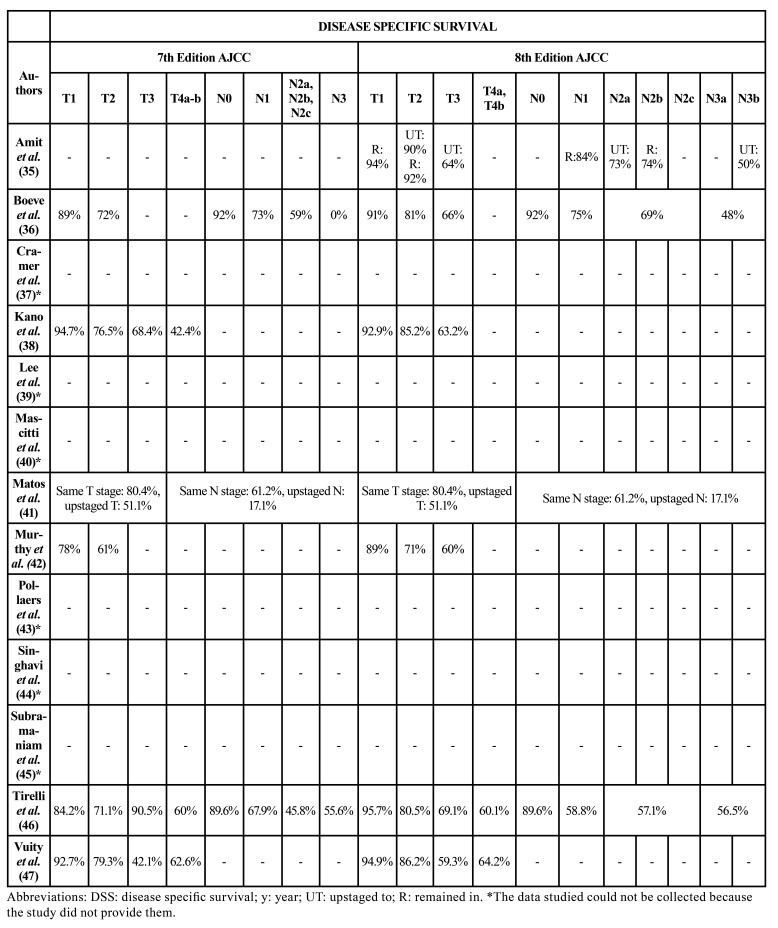
5-year disease specific survival by T and N 7th and 8th edition AJCC groups.

## Discussion

The overall results of the present study show that the new edition of the AJCC classification allows the choice of more correct treatment protocols and a better estimation of the prognosis in patients who previously were possibly undertreated - since an increase in classification is indicative of a poor prognosis, and such patients thus can receive more aggressive treatment to improve their survival.

From the publication of the first edition of the AJCC classification, the oral cavity and lips chapter experienced no significant changes up until the 6th edition of the classification (2002), where T4 lesions were divided into T4a (resecTable tumor) and T4b (unresecTable tumor) - resulting in the division of prognostic classification IV into classifications IVA, IVB and IVC. On the other hand, this edition did not include non-epithelial tumors such as those originating in soft tissue, lymphatic tissue, bone and cartilage. In the 7th edition (8), and in addition to the tumors excluded in the 6th edition, staging for melanoma of the mucosa of the lip and oral cavity was likewise not contemplated. The division of T4 classification remained in place, but T4a classification was defined as moderately advanced local disease, and T4b as very advanced local disease, likewise leading to the stratification of prognostic stage IV into IVA (moderately advanced local/regional disease), IVB (very advanced local/regional disease) and IVC (distant metastatic disease). Changes were then made to this classification with the aim of adapting to the need to classify patients correctly and thus facilitate an adequate treatment protocol and correct estimation of the prognosis - these being aspects that are maintained in the current edition of the classification (5).

The results of our systematic review show that most of the analyzed studies recorded poorer OS and DSS in patients who progressed to a higher T, N or prognostic stage than in those who remained in the same stage. On the other hand, patients showed similar or better OS or DSS when comparing the T, N or prognostic stage of the 8th edition versus the 7th edition.

The fact that only studies reporting survival rates at 5 years follow-up were included means that some articles had to be excluded, even if they could provide reliable data. However, the included studies involved similar methodology and their outcomes were compared at the same follow-up time, and this constitutes an important advantage. Another limitation of this study is that Lee *et al*. (39) and Cramer *et al*. (37) do an analysis on the same database. They show a difference between inclusion criteria of the patients: Lee *et al*. (39) only included patients that underwent definitive surgery, had not incomplete staging information and did not have metastasis; and Cramer *et al*. (37) include all patients. This is probably why they reach different conclusions.

Patients with similar OSCC classification may respond differently and have a different prognosis after receiving the same treatment, probably because the 7th edition of the classification and the conventional histopathological classification of tumors fail to predict the prognosis accurately, particularly in low-risk patients, despite being simple and consistent (35,46). The 8th edition of the AJCC classification, being the first to incorporate microscopic data in the staging of head and neck cancer, has attempted to address these problems by introducing the variables DOI and ENE. The fact that neither DOI nor ENE were included in the 7th edition (8) could imply that patients may have been incorrectly staged - with the consequent risk of under- or over-treatment.

Depth of invasion (DOI) assesses the invasiveness of OSCC independently of any exophytic or ulcerative component. To measure DOI, we must identify the line that marks the basement membrane of the adjacent mucosa. Once this is done, the distance from the mentioned line to the deepest point of invasion of the tumor millimeters (in mm) is measured by means of a perpendicular line. The second parameter (ENE) can be clinical or histopathological. Clinical ENE is defined as the extension of metastatic carcinoma in the lymph node through its capsule into the surrounding connective tissue, regardless of the existence of an associated stromal reaction. On the other hand, histopathological ENE can be assessed microscopically (ENEmi) or macroscopically (ENEma). The former is defined as extranodal extension visible to the naked eye at the time of dissection, while ENEmi is defined as microscopic extension over 2 mm beyond the capsule of the lymph node. If either of these two conditions is observed, the nodal status is indicated as positive (ENE+) (5).

In 2014, Ebrahimi *et al*. (48) proposed a modification for the 7th edition of the AJCC classification, after publishing a retrospective cohort study in which a statistically significant association was found between increased DOI and more advanced disease, including higher T and N classifications, extracapsular spread, and infiltrated margins. Thus, they proposed the incorporation of DOI into category T of the classification, which was later adopted by the AJCC classification in its 8th edition (5).

Many studies have also shown an increased DOI to be associated to an increased risk of neck lymph node metastases, and therefore to a poorer prognosis as well as to the presence of ENE (9). Similarly, Shaw *et al*. (49) found extracapsular nodal spread to be the most adverse histological predictor of disease recurrence in OSCC. Likewise, Wreesmann *et al*. (50) suggested that this scenario proves clinically relevant when extension exceeds 1.7 mm beyond the nodal capsule. Tirelli *et al*. (46) considered DOI to be an independent prognostic factor for survival, and reported significantly poorer DSS when DOI > 10 mm. In the same way, Matos *et al*. (41) found that patients with DOI > 10 mm suffered a higher prevalence of locoregional recurrence and distant metastases.

Most of the studies included in this systematic review found that the 8th edition of the AJCC classification increased the stage of many patients as a result of the new incorporations (ENE and DOI), in terms of T, N or prognostic stages, and that these individuals were therefore more correctly classified (35-41,43-46).

As shown by the results of the present review, the included articles obtained similar values for OS and DSS. Studies analyzing the new edition, comparing patients who were classified into a higher stage versus those who remained in the same group, found the former to have poorer survival (35,40,41,44). In particular, Almangush *et al*. (29) in a systematic review and metaanalysis published in 2021 found similar results as the current study: common upstaging of cases and good risk stratification of AJCC 8th edition for OSCC, which can change treatment planning of patients. On the other hand, authors comparing OS and DSS presenting data for each T, N or prognostic stage according to the 7th and 8th editions of the classification recorded higher survival rates using the latest edition (35-38,42,43,45-47). Therefore, the results obtained in this systematic review indicate that the 8th edition of the AJCC classification improves upon the previous edition in terms of prognosis (OS and DSS) among the T, N and prognostic stages.

## Conclusions

The introduction of the variables DOI and ENE in the 8th edition of the AJCC classification results in improved prognostic performance of the classification. Patients with OSCC can be better classified in relation to OS and DSS, while maintaining the simplicity and ease of use of the classification. This allows more appropriate treatment protocols to be applied, with a better estimation of the prognosis of each patient.
